# Well-differentiated prostate cancer in core biopsy specimens may be associated with extraprostatic disease

**DOI:** 10.1590/S1516-31802008000200010

**Published:** 2008-03-06

**Authors:** José Cury, Rafael Ferreira Coelho, Miguel Srougi

**Keywords:** Prostate, Prostate cancer, Prostatic neoplasms, Pathology, Pathology, surgical, Próstata, Câncer de próstata, Neoplasias prostáticas, Patologia, Patologia cirúrgica

## Abstract

**CONTEXT AND OBJECTIVE:**

Accurate determination of the Gleason score in prostate core biopsy specimens is crucial in selecting the type of prostate cancer treatment, especially for patients with well-differentiated tumors (Gleason score 2 to 4). For such patients, an inaccurate biopsy score may result in a therapeutic intervention that is too conservative. We evaluate the role of Gleason score 2-4 in prostate core-needle biopsies for predicting the final pathological staging following radical prostatectomy.

**DESIGN AND SETTING:**

Retrospective study at Hospital das Clínicas, Faculdade de Medicina da Universidade de São Paulo.

**METHODS:**

We analyzed the medical records of 120 consecutive patients who underwent radical retropubic prostatectomy to treat clinical localized prostate cancer at our institution between December 2001 and July 2006. Thirty-two of these patients presented well-differentiated tumors (Gleason score 2 to 4) in biopsy specimens and were included in the study. The Gleason scores of the core-needle biopsies were compared with the pathological staging of the surgical specimens.

**RESULTS:**

Sixteen of the 32 patients (50%) presented moderately differentiated tumors (Gleason score 5 to 7) in surgical specimens. Eighteen patients (56%) had tumors with involvement of the prostate capsule and ten (31%) had involvement of adjacent organs. Evaluating the 16 patients that maintained Gleason scores of 2 to 4 in the pathological staging of the surgical specimens, 11 (68.7%) had focal invasion of the prostate capsule and five (31.25%) had organ-confined disease.

**CONCLUSION:**

Well-differentiated tumors (Gleason score 2 to 4) seen in biopsies are not predictive of organ-confined disease.

## RESUMO

**CONTEXTO E OBJETIVO:**

A determinação acurada do escore de Gleason nas biópsias prostáticas é fundamental para seleção do tratamento adequado para o câncer de próstata, especialmente em relação aos tumores bem diferenciados (Gleason 2 a 4), para os quais a abordagem terapêutica pode ser mais conservadora. O objetivo deste trabalho foi avaliar a correlação entre o escore de Gleason 2 a 4 na biópsia de próstata com o estádio patológico final após a prostatectomia radical.

**TIPO DE ESTUDO E LOCAL:**

Estudo retrospectivo realizado no Hospital das Clínicas da Faculdade de Medicina da Universidade de São Paulo, São Paulo, Brasil.

**MÉTODOS:**

Foram revisados os prontuários médicos de 120 pacientes submetidos a prostatectomia radical retropúbica para tratamento de câncer de próstata localizado em nossa instituição entre dezembro de 2001 e julho de 2006. Trinta e dois destes pacientes apresentavam diagnóstico de câncer de próstata bem-diferenciado na biópsia prostática com agulha e foram incluídos no estudo. Os resultados das biópsias de próstata com agulha foram comparados com o estadiamento patológico final dos espécimes cirúrgicos obtidos com a prostatectomia radical.

**RESULTADOS:**

16 de 32 pacientes (50%) apresentaram câncer de próstata moderadamente diferenciado (escore de Gleason 5 a 7) na avaliação do espécime cirúrgico. 18 de 32 pacientes (56%) apresentavam acometimento da cápsula prostática pelo tumor, sendo que 10 (31%) destes pacientes apresentavam, em associação, envolvimento de órgãos adjacentes. Avaliando os 16 pacientes que mantiveram escore de Gleason 2 a 4 no estadiamento patológico da peça cirúrgica, 11 (68,7%) destes pacientes apresentavam invasão focal da cápsula prostática e 5 (31,25%) pacientes apresentavam doença confinada à próstata.

**CONCLUSÃO:**

Tumores bem diferenciados da próstata na biópsia com agulha (escore de Gleason 2 a 4) não são preditivos de doença órgão-confinada.

## INTRODUCTION

There is an international consensus that the Gleason system should be used for grading prostate cancer in histopathological specimens.^1,2^ This score, combined with the clinical stage and prostate-specific antigen (PSA) level, has strong prognostic significance and helps to determine the likelihood of cancer recurrence in patients who have undergone radical retropubic prostatectomy.^3,4^ Therefore, one major concern has been whether it is possible to predict the Gleason score from prostatectomy, from the limited samples obtained by means of core biopsies.^5^ This concept applies especially to patients with well-differentiated tumors for whom an inaccurate score may result in a more conservative therapeutic intervention, like watchful waiting, instead of surgery or radiotherapy.^6^ Some authors have proposed that Gleason scores of 2 to 4 should not be assigned to adenocarcinoma seen in needle biopsy material because it usually represents an undergrading of higher-grade carcinoma, is not accurately reproduced even by experts and may have an adverse impact on patient care.^7^ The issue of low-grade cancer seen in needle biopsy is one of the more controversial areas in urological pathology and deserves further evaluation.

Some previous studies have shown general concordance rates between the Gleason score from prostatectomy and the score obtained from core biopsies ranging from 28% to 74%.^8-19^ However, these studies included a great variation of Gleason scores from biopsies, ranging from undifferentiated tumors to low-grade cancer. In the present study, we evaluate the correlation between core biopsies and radical prostatectomy specimens among a specific subset of patients with well-differentiated tumors (Gleason score of 2 to 4) seen in core biopsies.

## OBJECTIVE

The aim of this study was to evaluate the role of Gleason score 2 to 4 in prostate core-needle biopsies for predicting the final pathological staging following radical prostatectomy.

## METHODS

We analyzed the medical records of 120 consecutive patients who underwent radical retropubic prostatectomy to treat clinical localized prostate cancer in our institution between December 2001 and July 2006. Thirty-two of these patients had well-differentiated tumors (Gleason score from 2 to 4) in biopsy specimens and were included in the study. The mean age, mean PSA level and clinical stage of these patients are described in Table 1.

**Table 1:** Characteristics of patients with well-differentiated tumors (Gleason score from 2 to 4) in biopsy specimens

**Mean age (years)**	62
**Mean PSA (ng/ml)**	5.9
**Clinical stage (n)**	
T1	22
T2	10

*PSA = prostate specific antigen.*

All the core biopsies were guided using transrectal ultrasonography (TRUS) with an end-fire 7 MHz probe and a spring-loaded core biopsy gun equipped with an 18 G needle. The biopsy protocol included six positions, of which three were on each side (sextant biopsies): apex, mid-medial and base. All biopsies were reviewed by three experienced pathologists and the Gleason score was determined for each patient.

The radical prostatectomy specimens were inked, fixed in 10% neutral formalin and serially sectioned at 3 mm intervals. The seminal vesicles were sectioned parallel to their junction with the prostate and subjected to examination in their entirety. The Gleason scores were obtained by summing the primary Gleason pattern grade and secondary Gleason pattern grade, based on assessment of the whole specimen. Surgical margins were considered to be positive when carcinoma cells were in contact with the inked margin. Invasion of the prostatic capsule, tumor extension to the prostatic apex or bladder neck and seminal vesicle involvement with carcinoma cells were considered to represent extraprostatic disease.

## RESULTS

The preoperative biopsies gave an exact prediction of the Gleason scores from radical prostatectomy, for 16 patients (50%). For the other 16 patients (50%), the biopsies undergraded the carcinoma, thus giving rise to Gleason scores of 5 to 7 (moderately differentiated) from radical prostatectomy (Figures 1A and 1B).

**Figure 1A:**
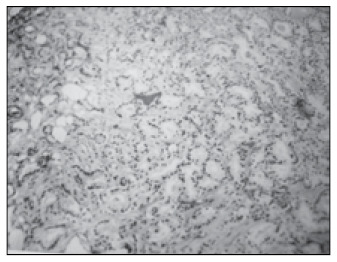
Gleason grade 1 pattern of prostate carcinoma from needle biopsy (Gleason score of 2 from biopsy).

**Figure 1B:**
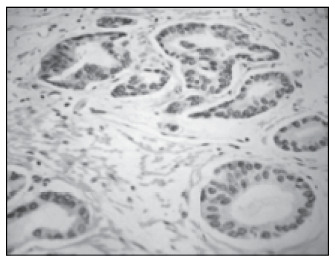
Surgical specimen from the same patient showing Gleason grade 3 pattern of prostate carcinoma (Gleason score of 6 from radical prostatectomy).

Only four (12.5%) of the 32 patients with well-differentiated carcinoma (Gleason score of 2 to 4) in the biopsy specimens had cancer confined to the prostate (negative surgical margins without extraprostatic disease) when the prostatectomy specimens were analyzed. Eighteen patients (56.25%) presented a focal extension to the prostatic capsule and six (19%) had extensive involvement of the prostatic capsule. Positive surgical margins in the bladder neck were found in eight patients (25%) and in the prostate apex in two (6.25%). The seminal vesicles were involved in two patients (6.25%) (Table 2).

**Table 2:** Extraprostatic involvement in radical prostatectomy specimens

Histopathological findings in prostatectomy specimens	n	Percentage (%)
Confined prostatic disease	4	12.5
Capsular infiltration	18	56.25
Positive margin in the bladder neck or apex	8	25
Seminal vesicle involvement	2	6.25
**Total**	**32**	**100**

Evaluating the 16 patients who maintained the Gleason score between 2 and 4 in the pathological staging of the surgical specimens, 11 (68.7%) of them had focal invasion of the prostate capsule and five (31.25%) had organ-confined disease.

## DISCUSSION

The Gleason grading system is widely used in urological pathology and has been adopted as a standard grading system by the World Health Organization (WHO).^20^ The Gleason grade is a powerful prognostic indicator for prostatic adenocarcinoma that has been validated by numerous studies. Its use is often critical for urologists, radiotherapists and oncologists in planning treatment.^21^ However, this system does have limitations. For example, the inter and intraobserver reproducibility varies among pathologists^22,23^ and the correlation between Gleason scores from biopsy and prostatectomy has been questioned in some previous studies. Exact concordance was found in 28% to 74% of the specimens from prostatectomy, while the biopsies undergraded the prostatectomy score in 24 to 60% and overgraded in 5 to 32%^8-19^ of the cases (Table 3). This inaccuracy of core biopsies in predicting the histopathological findings from specimens obtained via radical prostatectomy can clearly limit their clinical application and use in planning the treatment options. This concern applies especially to men with well-differentiated cancer and short life expectancy. For example, a 75-year-old man with a Gleason score of 4 and PSA of 6.5 may choose watchful waiting, whereas a Gleason score of 7 or higher may persuade him towards therapy that is more radical.^5^ Our study shows that 50% of the patients with well-differentiated tumors seen in core biopsies actually had moderately differentiated tumors (Gleason score 5 to 7). These patients might have chosen watchful waiting if the therapeutic approach had been selected based only on the Gleason grade from core biopsies.

**Table 3:** Correlation between Gleason scores from needle biopsy and radical prostatectomy

References	No. of patients	% concordance
Cury et al.^8^	120	32.5
Thickman et al.^9^	124	28
Garnett et al.^10^	115	30
Cookson et al.^11^	226	31
Bostwick^12^	316	35
Danziger et al.^13^	100	42
Paulson^14^	734	41
Fukagai et al.^15^	116	45.7
Mills and Fowler^16^	53	51
Spires et al.^17^	67	58
Steinberg et al.^18^	499	58
Carlson et al.^19^	106	68

The lack of Gleason score correlation between biopsies and prostatectomy specimens is partially explained by sampling errors.^24^ Prostate cancer has high morphological heterogeneity: over half of prostatectomy specimens contain cancer of at least three different Gleason grades, and cancer of a single grade is present in only 10-16% of specimens.^19-24^ Therefore, lack of representativeness is evidently a potential problem in prostate biopsy grading. Some of the discordance between biopsies also arises from intra and interobserver variability, as we mentioned before.^23,25,26^ Several systematic efforts have been made to reduce the observer variability in the Gleason grade system but some inter and intraobserver disagreement will always remain.^6,27^ We compared the interobserver variability between two experienced urological pathologists who analyzed the Gleason score of 45 prostate core biopsies from patients with adenocarcinoma of the prostate. The pathologists agreed on the Gleason score in only 45% of the cases.^8^

We believe that Gleason scores between 2 and 4 from prostate core biopsies should be carefully interpreted by clinicians. Assigning a Gleason score of 2 to 4 to adenocarcinoma from needle biopsies can adversely affect patient care if the clinicians assume that low-grade cancer does not need definitive therapy. When most tumors that are graded as Gleason score 2 to 4 by inexperienced pathologists are reviewed by experts, they are graded as Gleason score 5 to 6 or higher.^18^ Furthermore, the reproducibility of Gleason scores of 2 to 4 is poor even among urological pathology experts.^22^ Consequently, some men with tumors diagnosed from needle biopsy, in which the assigned grade is Gleason score 2 to 4, will potentially be undertreated or at least be improperly counseled about the risk of tumor progression if expectant therapy is selected. The assurance that a given tumor is indolent, based on a low Gleason score from needle biopsy, is not well-founded. Indeed, our evaluation of surgical specimens showed that 56% of the patients with low-grade cancer, as seen in core biopsies, presented tumor involvement in the prostate capsule and 31% had involvement of adjacent organs. Steinberg et al.^18^ evaluated 87 needle biopsies with cancers graded as Gleason score 2 to 4 by pathologists from other institutions, and showed, through radical prostatectomy, that 48 (55%) of the cases had extraprostatic extension, including four cases with invasion of either seminal vesicles or lymph nodes.

Recently, some prognostic models have been developed to predict the likelihood of a given tumor being at specific pathological stage more accurately than by using the core biopsy and Gleason score separately. In a multi-institutional study, Partin et al.^28^ examined clinical and pathologic data from 4133 men who underwent radical retropubic prostatectomy. Serum PSA level, tumor type, TNM clinical staging of the metastasis and Gleason score were identified as significant predictors of pathological stage. Similarly, Kattan et al.^29^ combined clinical prognostic factors to predict the likelihood of biochemical disease recurrence following radical prostatectomy. By combining serum PSA, TNM clinical stage and Gleason score from biopsy, a nomogram that predicted the five-year likelihood of treatment failure among men with clinical localized prostate cancer treated with radical prostatectomy was developed. These prognostic models should enable patients and physicians to make better-informed treatment decisions on the basis of the patient’s clinical situation. Although Gleason grade is an important prognostic factor, we believe that it cannot be used categorically to determine the prognosis or to justify the management.

Some proposals to improve the concordance of grading prostate needle biopsies and radical prostatectomy specimens have also been studied. Extended prostate needle biopsies (10 or more cores), for example, have been evaluated in some studies with discordant results. San Francisco et al.^30^ found that extended biopsies had greater accuracy for predicting the final Gleason score (76% of extended biopsies produced identical Gleason scores versus 67% in non-extended biopsies) and concluded that extended prostate needle biopsies provided better guidance for determining the appropriate treatment for prostate cancer patients. On the other hand, Thickman et al.^9^ studied 124 biopsies and reported that increasing the number of cores beyond six did not improve the concordance. Egevad et al.^5^ observed only a marginal improvement in prediction of the Gleason score from prostatectomy through increasing the number of biopsies (the accuracy increased from 43.5% to 45.2%). Another strategy proposed for improving the reliability of needle biopsy grading was repetition of the TRUS biopsy in patients with well-differentiated tumors.^5^ Fleshner et al.^6^ studied patients with Gleason scores of 6 or less from prostate biopsies. They found that 38% of these patients had final pathological grades of 7 or more when they underwent to a single biopsy. Among the patients with well-differentiated tumors who underwent two prostate biopsies, only 19% had final pathological grades of 7 or more. They concluded that prostate re-biopsy can minimize the unreliability of the Gleason grade from biopsies and should particularly be considered for patients with well-differentiated tumors who choose watchful waiting or for patients for whom upgrading would result in a change to the therapeutic intervention.

## CONCLUSIONS

Well-differentiated prostate tumors (Gleason score 2 to 4) seen in core biopsies are not predictive of organ-confined disease. The reality, from evaluating surgical specimens, was that 50% of the patients with well-differentiated tumors seen in core biopsies had moderately differentiated tumor (Gleason score 5 to 7), 56% had involvement of the tumor with the prostate capsule and 31% had involvement with adjacent organs. This inaccuracy of core biopsies in relation to predicting the histopathological findings from radical prostatectomy specimens can clearly limit the clinical application and utility of core biopsies for planning treatment options.
